# Long-Term Dynamics Among *Wolbachia* Strains During Thermal Adaptation of Their *Drosophila melanogaster* Hosts

**DOI:** 10.3389/fgene.2020.00482

**Published:** 2020-05-14

**Authors:** Rupert Mazzucco, Viola Nolte, Thapasya Vijayan, Christian Schlötterer

**Affiliations:** ^1^Institut für Populationsgenetik, Veterinärmedizinische Universität Wien, Wien, Austria; ^2^Vienna Graduate School of Population Genetics, Vienna, Austria

**Keywords:** experimental evolution, evolve-and-resequence, strain frequency, copy number, microecology, microbe–host interaction, DCV resistance

## Abstract

Climate change is a major evolutionary force triggering thermal adaptation in a broad range of species. While the consequences of global warming are being studied for an increasing number of species, limited attention has been given to the evolutionary dynamics of endosymbionts in response to climate change. Here, we address this question by studying the dynamics of *Wolbachia*, a well-studied endosymbiont of *Drosophila melanogaster*. *D. melanogaster* populations infected with 13 different *Wolbachia* strains were exposed to novel hot and cold laboratory environments for up to 180 generations. The short-term dynamics suggested a temperature-related fitness difference resulting in the increase of clade V strains in the cold environment only. Our long-term analysis now uncovers that clade V dominates in all replicates after generation 60 irrespective of temperature treatment. We propose that adaptation of the *Drosophila* host to either temperature or *Drosophila* C virus (DCV) infection are the cause of the replicated, temporally non-concordant *Wolbachia* dynamics. Our study provides an interesting case demonstrating that even simple, well-controlled experiments can result in complex, but repeatable evolutionary dynamics, thus providing a cautionary note on too simple interpretations on the impact of climate change.

## Introduction

The global change in climate imposes strong pressure on many species to deal with increasing temperatures (Thuiller et al., 2005; Cheung et al., 2009; Klausmeyer and Shaw, 2009)—using either mitigation strategies (e.g., shifts in range or activity periods; Davis, 2001; Menzel et al., 2006; Chen et al., 2011), or through genetic changes (e.g., thermal adaptation; Calosi et al., 2008; Marshall et al., 2010; Somero, 2010; Hoffmann and Sgrò, 2011). In the presence of endosymbiotic bacteria, adaptation to temperature could occur by genetic changes either in the host or bacteria—even co-evolutionary processes between both of them could contribute to thermal adaptation.

*Wolbachia* are intracellular α-Proteobacteria found in many insect and other arthropod species (Baldo et al., 2006; Mateos et al., 2006; Werren et al., 2008), infecting about two thirds of all insects (Hilgenboecker et al., 2008; Miller, 2013). They are predominantly transmitted through the female germline and often confer fitness advantages; e.g., virus protection (Hedges et al., 2008; Chrostek et al., 2013; Faria et al., 2018), learning ability (Bi et al., 2018), increased fecundity (Fry et al., 2004), resistance to heat stress (Gruntenko et al., 2017), and influence longevity (Maistrenko et al., 2016). On the other hand, *Wolbachia* frequently also imposes considerable costs on its host through the reduction in effective population size by male-killing (Hurst et al., 1999), feminization of genetic males (Rigaut, 1997) and cytoplasmic incompatibility (Bourtzis et al., 1996; Hoffman and Turelli, 1997). In addition to fitness effects of *Wolbachia* on its host, the fitness of the infected host and the probability of vertical transmission also affect the fitness of *Wolbachia*. Among the factors contributing to these fitness components are temperature (Jia et al., 2009; Bordenstein and Bordenstein, 2011), bacterial density in the host (Breeuwer and Werren, 1993; Bourtzis et al., 1996; Noda et al., 2001), and the genetic background of the host (Olsen et al., 2001; Reynolds and Hoffmann, 2002; Fry et al., 2004).

Multiple strains—sometimes several supergroups—of *Wolbachia* may compete within a host population (Dean et al., 2003; Mouton et al., 2003). While coinfection and thus competition within single hosts has been described (Fleury et al., 2000; Hiroki et al., 2004; Ant and Sinkins, 2018), competition mainly occurs between hosts. The relative fitness of multiple *Wolbachia* strains can be measured by the spread of the fitter strain(s) in sexual populations. A particularly interesting question is how the fitness of different *Wolbachia* strains is affected by the environment.

A pioneering study used experimental evolution to study temperature adaptation by exposing a replicated polymorphic *Drosophila melanogaster* population infected by multiple *Wolbachia* strains to two different temperature regimes (Versace et al., 2014). The dynamics of *Wolbachia* infection were monitored by clade-specific SNPs in Pool-Seq data (Schlötterer et al., 2014) from up to four replicates at multiple time points in hot and cold temperature regimes. The striking result was that in the cold temperature regime *Wolbachia* from a single clade (V) very rapidly predominated. Even in hot-evolved replicates that were shifted to the cold temperature regime the same *Wolbachia* clade V dominated. This consistent association of clade V with cold temperatures was considered strong support for environmentally triggered fitness differences between *Wolbachia* strains. Here, we extend the previous work by characterizing the *Wolbachia* dynamics on the level of individual strains rather than clades and our analyses cover substantially more generations in more replicates.

## Materials and Methods

### *Drosophila melanogaster* Population and Culture Conditions

We reanalyze an evolve-and-resequence experiment (Turner et al., 2011) for which allele frequency changes in *D*. *melanogaster* (Orozco-terWengel et al., 2012; Tobler et al., 2014; Franssen et al., 2015), and *Wolbachia* strain turnover during the first 50 generations were reported (Versace et al., 2014); detailed descriptions of the experimental setup can be found there. Briefly, 10 replicate populations each with approximately 1000 individuals were created from 113 *D. melanogaster* isofemale lines collected in Portugal and were subsequently kept in two different temperature regimes: five replicates in a hot environment fluctuating between 18 and 28°C, and five replicates in a cold environment fluctuating between 10 and 20°C. Of the 113 isofemale lines, 47 were known to carry *Wolbachia.* The 10 replicates at generation 0 are considered as the base population.

The previous datasets included only time points from the early phase of the experimental evolution cages. Here, we extend the analyses to advanced phases of the experiment by including additional time points and replicates for both the hot and the cold evolved populations: while Orozco-terWengel et al. (2012) and Versace et al. (2014) analyzed up to three replicates in the hot evolved populations until generation F37, we now include data for up to five replicates at multiple earlier and later time points until generation F180 in the hot environment. For the cold environment, Versace et al. (2014) analyzed four replicates in generation F15. Here, we add the fifth replicate and multiple time points up to generation F100 (Supplementary Datasheet S2).

### Sequencing and Postprocessing

Single females of each of the 47 isofemale lines infected with *Wolbachia* were sequenced individually (2 × 100 bp; ∼10–30× autosome coverage). The infection status of the isofemale lines had been previously determined using the protocol described below (section “Confirmation of *Wolbachia* Infection Status”). Pools of flies (Kofler et al., 2011; Schlötterer et al., 2014) were sequenced at different time points over the course of the experiment, including three replicates of the base population (∼500 flies per generation and replicate; paired-end; ∼30× autosome coverage; various read lengths, library preparation protocols, providers, and sequencing platforms following the development of Illumina sequencing over a decade; Supplementary Datasheet S2).

Reads were trimmed with ReadTools v1.2.1 (Gómez-Sánchez and Schlötterer, 2017; parameters: –disable5pTrim –mottQualityThreshold 20 –minReadLength 34); mapped with novoalign v3.08 (Novocraft, 2018; parameters: -r RANDOM) and bwa v0.7.17 (Li, 2013; parameters: mem) using our standard DistMap pipeline (Pandey and Schlötterer, 2013) against the combined reference genome of *D. melanogaster* v6.03 (Thurmond et al., 2019), *w*Mel (AE017196.1), and common gut bacteria (Petkau et al., 2016; *Acetobacter pasteurianus*, AP011170.1; *Lactobacillus brevis*, CP000416.1; *Lactobacillus plantarum*, CP013753.1); filtered for quality and overlap with the *w*Mel genome or the mtDNA genome with samtools v1.9 (Li et al., 2009; parameters: -f0x02 -q 5); and had duplicates removed with picard v2.12.1-SNAPSHOT (The Broad Institute, 2018; parameters: MarkDuplicates REMOVE_DUPLICATES = true).

### Variant Calling and Marker Sites

Variants in the 47 sequenced individuals were called using freebayes v1.2.0 (Garrison and Marth, 2012; parameters: -p2 –pooled-discrete) using the alignments of novoalign and bwa jointly to account for the mapper-specific influence of insert size differences on SNP calling (Kofler et al., 2016). Among the resulting variants, we selected SNPs that only occurred in a true subset of the 47 samples, i.e., allow to discern among strains, and met minimum coverage and quality criteria using bcftools v1.9 [Li, 2011; parameters: -i ‘TYPE = “SNP” & INFO/DP < 3^∗^mean(DP) & NS = 47 & NUMALT = 1 & QUAL > 40 & MQM > 50 & MQM/MQMR > 4/5 & MQM/MQMR < 5/4 & RPL/RPR > 1/3 & RPL/RPR < 3 & SAF/SAR > 1/3 & SAF/SAR < 3 & SRF/SRR > 1/3 & SRF/SRR < 3’ -e ‘FORMAT/GT! = “hom,”’ where “mean(DP)” in the first condition is the mean depth of all 47 samples over all sites calculated beforehand, and separately for *Wolbachia* and mtDNA contigs], leaving us with 197 high-quality marker SNPs to discern among the *Wolbachia* strains present in the 47 infected founder lines (Supplementary Datasheet S3: markers_wmel.vcf.gz) and 29 marker SNPs to discern among the mitochondrial clades (Supplementary Datasheet S3: markers_mtDNA.vcf.gz). Not all SNPs are equally informative, being shared by two or more strains.

### Strain and Clade Identification

**FIGURE 1:**
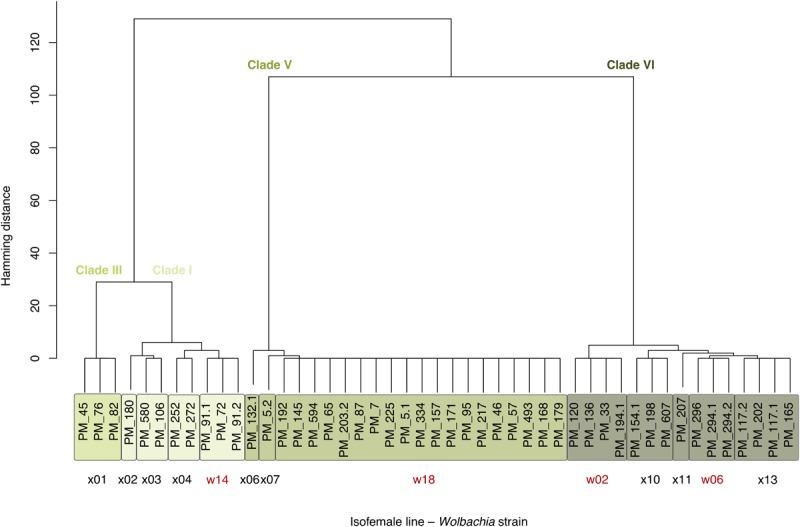
47 Isofemale lines of the *D. melanogaster* base population carry 13 *Wolbachia* strains representing four clades. The tree shows the isofemale lines clustered by Hamming distance *h* over 197 high-quality marker SNPs on the *w*Mel genome. The three clusters at *h* = 29 correspond to the *Wolbachia* superclades discussed in Versace et al. (2014), where also strains w02, w06, w14, and w18 (red) were introduced. The four clusters at *h* = 6 (color labels) correspond to the *Wolbachia* clades as defined in Richardson et al. (2012). Clade VI strains resemble the variant also known as *w*MelCS.

Based on the polymorphic sites (markers), we distinguished 13 *Wolbachia* strains (Figure 1), 10 of which have private SNPs. Comparing the markers to the strain-specific SNPs identified previously (Versace et al., 2014), we identify the same overall clade structure with some additional, previously unresolved, fine structure. Accordingly, we continue using the same naming convention. This assignment is fully consistent for *Wolbachia* and mitochondria strains to clades based on the SNPs provided in Richardson et al. (2012), where the clade structure was originally established.

### Estimation of Strain Frequencies

SNP frequencies in all Pool-Seq samples at the previously detected marker sites were called with freebayes (parameters: -F 0.01 -C 2 –pooled-continuous). Given markers for *n* strains and a Pool-Seq sample with reference allele frequencies *b* at *m* marker sites, we estimate the *n*-vector of corresponding strain frequencies *x* by minimizing w|*Ax-b*| subject to constraints 0 < *x*_*j*_ < 1 and ∑*x*_*j*_ = 1 with the conjugate-gradient method, in which *A* is an *m* × *n*-matrix with columns containing 1 where the corresponding strain is marked by the reference allele and 0 otherwise, *b* an *m*-vector of called reference allele frequencies, and *w* an *m*-vector containing the coverage depth at the marker sites, serving as weights to the minimization procedure.

Ideally, only SNPs private to a strain (i.e., that have a multiplicity of 1) should be used to estimate the frequency of a given strain as the median over the frequencies of the strain-specific SNPs. However, private SNPs are not available for all strains. We thus use the most informative subset of marker sites large enough to differentiate among all strains, obtained by iteratively removing the marker sites with the highest multiplicity until the Shannon entropy (Shannon, 1948) per matrix row is maximized. This retains 75 of the 197 SNPs that differentiate *Wolbachia* strains, 120 of the 180 SNPs that differentiate clades, and all 155 SNPs that differentiate superclades, as well as all 21 mtDNA SNPs that differentiate mtDNA superclades. Since the median is the central point that minimizes the mean absolute deviation, our procedure is equivalent to median estimation when used with only private SNPs and equal weights (Stepniak, 2016). Code is provided in Supplementary Datasheet S4.

### Confirmation of *Wolbachia* Infection Status

While Versace et al. (2014) demonstrated that all flies were infected with *Wolbachia* within less than 37 generations in either temperature regime, the infection could be lost at later generations. We therefore confirmed the infection status at the final generation (F100 in the cold, F180 in the hot environment) via PCR of at least 30 individual male flies per replicate. We extracted DNA using a salting-out procedure (Miller et al., 1988). To determine the *Wolbachia* infection status, we performed PCR using primers wsp81F (5′-TGGTCCAATAAGTGATGAAGAAAC-3′) and wsp691R (5′-AAAAATTAAACGCTACTCCA-3′) (Braig et al., 1998) resulting in a 630 bp fragment of the *Wolbachia wsp* gene. To rule out that the absence of a *wsp* PCR fragment was due to low quality DNA or suboptimal PCR conditions, we chose primers LV125-F (5′-GAGTCGGTTTCCCACAAAG-3′) and LV125-R (5′-GAGCACATCTACGAGTTTCC-3′) to amplify in parallel a 349 bp fragment of *D. melanogaster* DNA in the same PCR reaction. PCR amplifications were performed in 20 μl reaction volumes using 2.5 mM MgCl_2_, 0.2 mM dNTPs, 10 pmol of each primer, 0.4 U FIREPol *Taq* Polymerase in buffer B (Solis Biodyne, Tartu, Estonia), and ca. 10 ng genomic DNA. PCRs were run under the following conditions: 3 min at 94°C for initial denaturation followed by 32 cycles of 94°C for 30 s, 55°C for 30 s, 72°C for 50 s, and a final extension step of 72°C for 7 min. In a few samples with weak or absent amplification of the *wsp* PCR fragment, an additional PCR with *Wolbachia*-specific primers (wMel-clades_fw: 5′-CACTTTTCTGCTGCTGTTATAC-3′, wMel- clades_rv: 5′-AGAGGGTATTTATGGTAGCAAG-3′) was used with the same conditions to verify the presence or absence of *Wolbachia*.

### Copy Number Estimation

We estimated *Wolbachia* and mitochondrial copy numbers from the coverage depth of the *Wolbachia*, or mitochondria, genome relative to the coverage depth of the *Drosophila* autosomes to account for read depth heterogeneity among libraries. Since the low GC content results in a systematic underestimation of read coverage, we corrected for GC bias by GC matching: all positions in the reference genome are assigned an effective GC content, defined as the average GC content of a DNA fragment that covers this position, and calculated as a weighted count of GC bases around the focal position, with weights constructed from the estimated read length and insert size distributions. Positions are then binned by GC content. The copy number is obtained as a weighted mean over GC bins of the relative coverage depths on the target contigs (*w*Mel, mtDNA) and normalization contigs (all *Drosophila* autosomes), with weights *mn/(m+n)* accounting for the number of positions *m* on the target contig and *n* on the normalization contigs within each GC bin (Supplementary Datasheet S4). Copy numbers are given as copies per host cell.

## Results

Based on 197 informative polymorphisms, we distinguished 13 distinct *Wolbachia* strains from 47 *Wolbachia*-infected isofemale lines. These 13 strains cluster into three major groups and can be assigned to clades I, III, V, and VI as defined by Richardson et al. (2012). No strain belonging to clade II or clade IV was identified (Figure 1).

**FIGURE 2:**
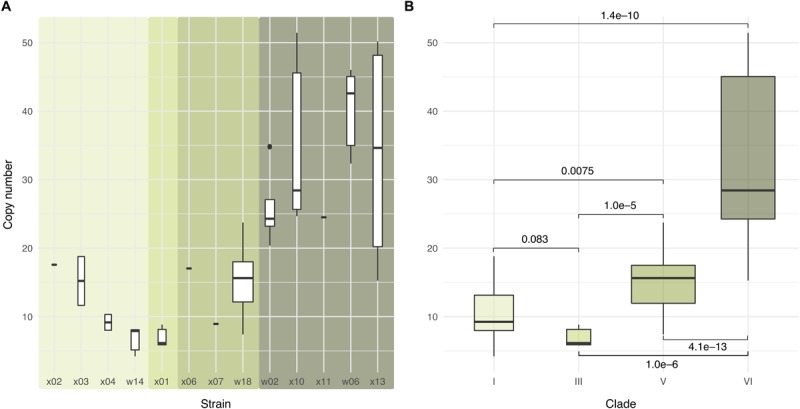
*Wolbachia* copy numbers differ substantially among strains. Box plots in the upper row show *Wolbachia* copy numbers in representative individuals from the 47 isofemale lines **(A)** by strain and **(B)** by clade, with boxes indicating the two middle quartiles of the distribution and whiskers extending to the largest or smallest value no further than 1.5 times the inter-quartile range from the hinge. Background shading in **(A)** indicates the clades depicted in **(B)**. Brackets labels in **(B)** show the *p-*values from a two-sided Wilcox test for the null hypothesis of the same mean copy number (Supplementary Table S1, also for strains).

Previous studies reported variation in copy number between different *Wolbachia* strains (Min and Benzer, 1997; Ijichi et al., 2002; Salzberg et al., 2005; Chrostek et al., 2013). Grouping the 47 samples into four clades confirmed pronounced differences. The highest copy number was seen in clade VI and the lowest in clade III. The copy numbers of clade V and VI, in particular, are clearly differentiated (Figure 2).

The relative frequency of the *Wolbachia* strains is obtained from Pool-Seq data by taking advantage of the strain/clade specific SNPs. Nevertheless, this method is not informative about the fraction of flies being infected. Versace et al. (2014) tested individual flies and found that at generation 37, all flies tested were infected. We confirm the infection status after long-term evolution (generations 100 in the cold and 180 in the hot environment). With at least 30 sampled flies from each replicate, we conclude that the infection status did not change between generation 37 and generations 100 or 180 (Supplementary Table S2).

**FIGURE 3:**
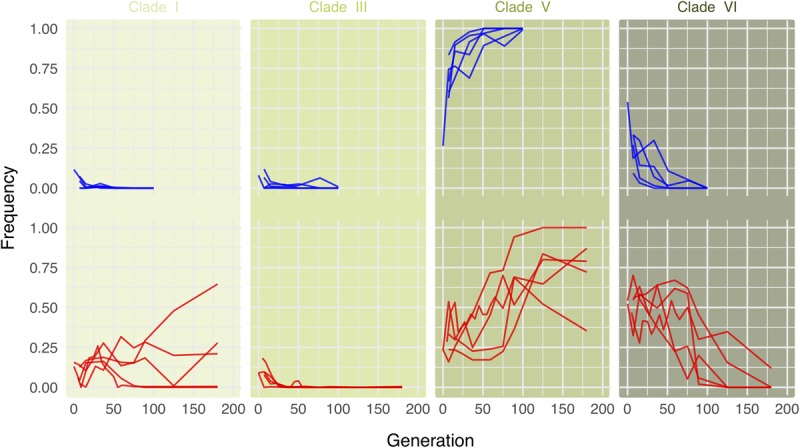
Long-term dynamics reveal that the same *Wolbachia* clades are successful in both hot and cold environments. Lines show clade frequencies over time for five replicates each in either the cold (upper row; blue) or the hot (lower row; red) environment, based on the most informative 120 of the 180 marker SNPs differentiating among clades. Clade V is consistently more successful in the long term in either environment.

Consistent with the results of Versace et al. (2014) we find that in the cold environment, clade V very quickly replaces the other *Wolbachia* clades (Figure 3, upper row). In the hot environment, a different pattern is observed. In all five replicates, clade VI is predominant during the first generations, as already noted in Versace et al. (2014). Starting around generation 70, however, the same clade V that dominates in the cold environment replaces the other *Wolbachia* genotypes (Figure 3, lower row, Supplementary Figure S1, and Supplementary Table S3). Owing to the delayed response, the anti-correlation between clade V and clade VI is a bit weaker in the hot environment (Supplementary Figure S2). This long-term behavior differs from expectations based on the results of Versace et al. (2014), who only studied the dynamics in the hot environment until generation 37.

**FIGURE 4:**
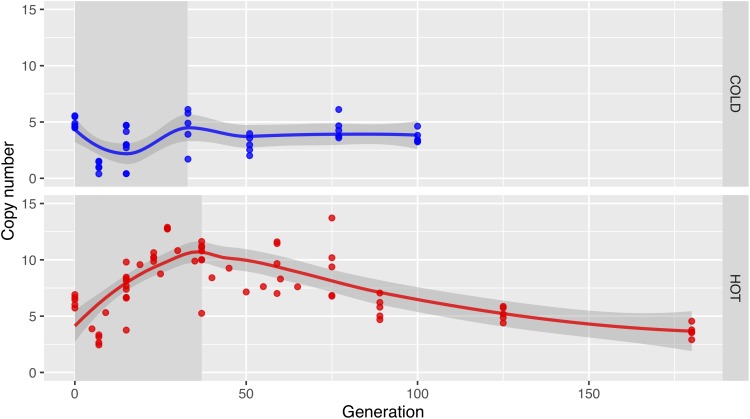
In the long term, *Wolbachia* copy numbers decrease in the hot environment. Dots show the *Wolbachia* copy numbers in replicates estimated with GC matching from the relative coverage depths in cold (upper row; blue) and hot (lower row; red) environments, the lines visualize the main trend obtained by Loess-smoothing. Only a fraction of the population was infected before generation 33 in the cold environment or generation 37 in the hot environment (gray background shading).

The dynamics of *Wolbachia* strain turnover are also reflected in the mean *Wolbachia* coverage at the different time points. In the hot environment, we first observe an increase in coverage, which reflects mainly the increasing infection frequency. After generation 37, the copy number drops, reflecting the taking over of low-copy-number strains (Figure 4 and Supplementary Figure S3). In the cold, the infection frequency also increases, but unlike the hot environment, this does not result in a higher coverage, because the low-copy number strains predominate already at the early generations. Rather, we notice first a drop in coverage, followed by a recovery of the coverage as the entire population becomes infected with *Wolbachia* until generation 33 (Versace et al., 2014).

**FIGURE 5:**
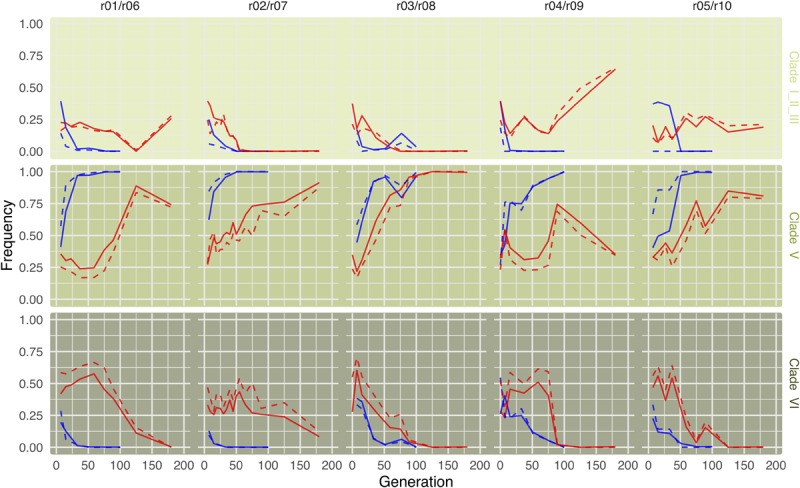
Mitochondrial dynamics on the superclade level are consistent with *Wolbachia* dynamics. Lines indicated superclade (as defined by Versace et al., 2014) frequencies for both mitochondria (solid lines) and *Wolbachia* (dashed lines) in cold (blue) and hot (red) environments. While frequencies are based on 155 marker SNPs differentiating among the three superclades, mitochondria frequencies are based on only 21 differentiating marker SNPs (owing to their smaller genome). The grouping of two replicates from different temperature treatments in a panel is random. All replicates evolved independently.

Given the high consistency of the phylogenetic relationship of mtDNA and *Wolbachia* seen in 290 *melanogaster* lines (Richardson et al., 2012), we expected that the *Wolbachia* dynamics are mirrored by the mtDNA dynamics. A direct comparison is, however, complicated by the lower number of SNPs in the mtDNA, resulting in a lower resolution and more noise. Therefore, we compared the dynamics on the level of super clades, as defined by Versace et al. (2014), which combines clade I, II, and III. Consistent with our expectation, we find an excellent overall correlation between *Wolbachia* and mitochondria (Supplementary Figure S4). We attribute the minor deviations to difficulties with an unambiguous clade assignment, rather than biological differences (Figure 5).

**FIGURE 6:**
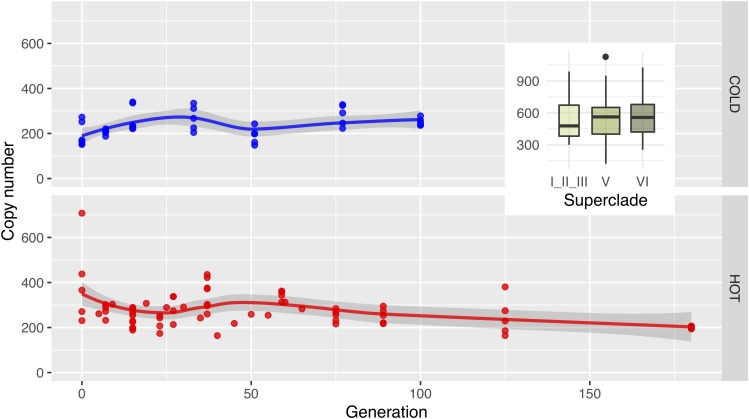
Mitochondrial copy numbers remain constant. Dots show the mitochondrial copy number for all replicates in cold (upper row; blue) and hot (lower row; red) environments, while the lines visualize the main trend via Loess-smoothing. Copy numbers were estimated with GC matching from the relative coverage depths. Boxplots in the insert show the copy numbers of mitochondria in the isofemale lines on superclade level (compare Figure 2; *p-*values from a Wilcox test in Supplementary Table S1).

Like *Wolbachia* copy number, we also evaluated whether mtDNA copy numbers change during the experiment. Unlike *Wolbachia*, the mtDNA copy number is very stable. This observation is fully consistent with the very similar copy numbers in all strains analyzed (Figure 6).

## Discussion

Compared to Versace et al. (2014), this study covers three advancements. First, we increased the number of replicates and show that the results of Versace were robust. Second, we provide a full SNP catalog of all *Wolbachia* strains in the experiment. This analysis showed that multiple different strains contribute to the clade specific dynamics previously described. Hence, *Wolbachia* strains belonging to the same clade are behaving similarly in their evolutionary response. Third, we increase the number of generations by more than fourfold. While the long-term dynamics in the cold environment do not change, we notice an interesting difference in the hot environment. While during the first generations the turnover of *Wolbachia* genotypes is rather modest, at later generations the same clade V that predominates in the cold cage outcompetes all other ones in the hot cage.

The temporal inconsistency of the evolutionary response cannot be explained by stochastic changes, as it is observed in all five replicates—albeit with different dynamics. The dynamics of the *Wolbachia* strains is not consistent with temperature being the only factor determining the frequency of the *Wolbachia* strains in the evolving replicates. Because the temperature regime is not changing over time, a consistent trend would have been expected. We observe, however, that the early phase differs from later time points. In the following, we will discuss some scenarios, which may explain the repeatable pattern of temporal heterogeneous *Wolbachia* dynamics.

The first hypothesis is that some uncontrolled environmental variables have changed during the experiment. This may include slight modifications in the food, due to different suppliers or modification in the maintenance protocol. As evolved flies are more fecund than ancestral ones from the base population (e.g., Barghi et al., 2019), egg laying time was reduced and larval density may also have changed.

The second hypothesis assumes that adaptation of the host affected the dynamics of the different *Wolbachia* strains, as has been shown for other stocks maintained in the laboratory (Correa and Ballard, 2014). Given that our experiment was designed to study the impact of temperature, adaptation of *Drosophila* to the new temperature regime may explain the dynamics. In a similar experiment, *Drosophila simulans* has been shown to have phenotypically converged at generation 60 (Barghi et al., 2019). Thus, it may be possible that different *Wolbachia* strains may be favored before and after the flies reaching trait optimum.

The third hypothesis is motivated by the observation that the evolved populations sometimes showed symptoms that are typical hallmarks of *Drosophila* C virus (DCV) infection (black, elongated, dying larvae and pupae; Ashburner and Roote, 2007). We propose that the dynamics may relate to the impact of *Wolbachia* copy number on host fitness in the presence of the DCV. A high *Wolbachia* copy number has been shown to be favorable in DCV infected flies when clade VI was compared to clades I, II, and III (Chrostek et al., 2013). Consistent with this, in a DCV-challenged population infected with clades I, II, III, and V, *Wolbachia* of the clade with the higher copy number (V) increased relative to clade I/III, but in the control population, no change was observed (Faria et al., 2016). A particularly interesting feature of our evolving populations is that, for the first time, two high-copy clades, V and VI, can be directly compared against each other. Because in our experiment clade VI has the highest copy number (Figure 2), this *Wolbachia* strain should provide the highest protection against DCV, but it is outcompeted by clade V. We attribute this apparent discrepancy to the fitness costs caused by high *Wolbachia* copy numbers (Fleury et al., 2000; Fry et al., 2004; Zhukova and Kiseleva, 2012; Chrostek and Teixeira, 2015; Martinez et al., 2015).

Another explanation for the increase of clade V is that if the *Drosophila* host responds to this DCV challenge by developing resistance, the advantage of the high copy *Wolbachia* may be diminished and another *Wolbachia* strain with lower copy number may take over. We addressed this hypothesis and analyzed the dynamics of two sequence variants, which confer DCV resistance in *Drosophila* (Martins et al., 2014) as an indicator of the resistance level of the *Drosophila* host. The resistance allele of the *pastrel* locus occurs at very low frequencies only and does not respond during the experiment (Supplementary Figure S5). The second resistance allele increases in some replicates, but not in all. While it is possible that other DCV resistance loci contribute, we do not have strong evidence for the *Drosophila* host developing DCV resistance during the experiment. This implies that if the evolving populations were challenged by DCV and developed strategies against DCV, this has been mainly achieved by changing the *Wolbachia* strain composition—a hypothesis that could be experimentally tested in future studies.

Finally, as a fourth hypothesis, *Wolbachia* may have adapted to their new environment by the acquisition of new mutations. We consider this highly unlikely because in all five replicates the same three *Wolbachia* strains increased in frequency in the hot environment at the later generations. This would require that all three, highly similar, strains independently acquired new mutations providing a fitness advantage. Furthermore, one would need to find additional explanations, such as epistasis, for the observation that only a single clade increases in frequency at the later generations. Finally, we did not detect new mutations in these strains that could explain the increase in fitness (data not shown).

Independent of the actual cause for the changes in *Wolbachia* dynamics, our study demonstrated that long-term experimental evolution may uncover evolutionary dynamics that remain unnoticed in short-term experiments. Particularly interesting would be further work to illuminate the influence of the host genotype on the observed *Wolbachia* dynamics.

As the hot environment was found to have short- and long-term dynamics, our experiments also highlight the difficulty in making predictions about the impact of temperature changes, thus providing a cautionary note on too simple interpretations on the impact of climate change.

## Data Availability Statement

All short-read data used in this study are available from the European Nucleotide Archive (PRJEB37761), but have in parts also been made available earlier (see Supplementary Datasheet S2 for details). Variant data are included as Supplementary Datasheet S3.

## Author Contributions

CS designed the study. VN conducted the experiments. TV performed a subset of the PCR measurements. RM performed the analysis. RM and CS wrote the manuscript. All authors contributed to manuscript revision and approved the submitted version.

## Conflict of Interest

The authors declare that the research was conducted in the absence of any commercial or financial relationships that could be construed as a potential conflict of interest.
