# Supplementary material for: Long-Term Dynamics Among Wolbachia Strains During Thermal Adaptation of Their Drosophila melanogaster Hosts
Source: Front Genet. 2020 May 14;11:482. doi: 10.3389/fgene.2020.00482 (PMC7241558; doi:10.3389/fgene.2020.00482)
Supplement: Supplementary file 1 [file Data_Sheet_1.PDF]

Supplemental figures and tables

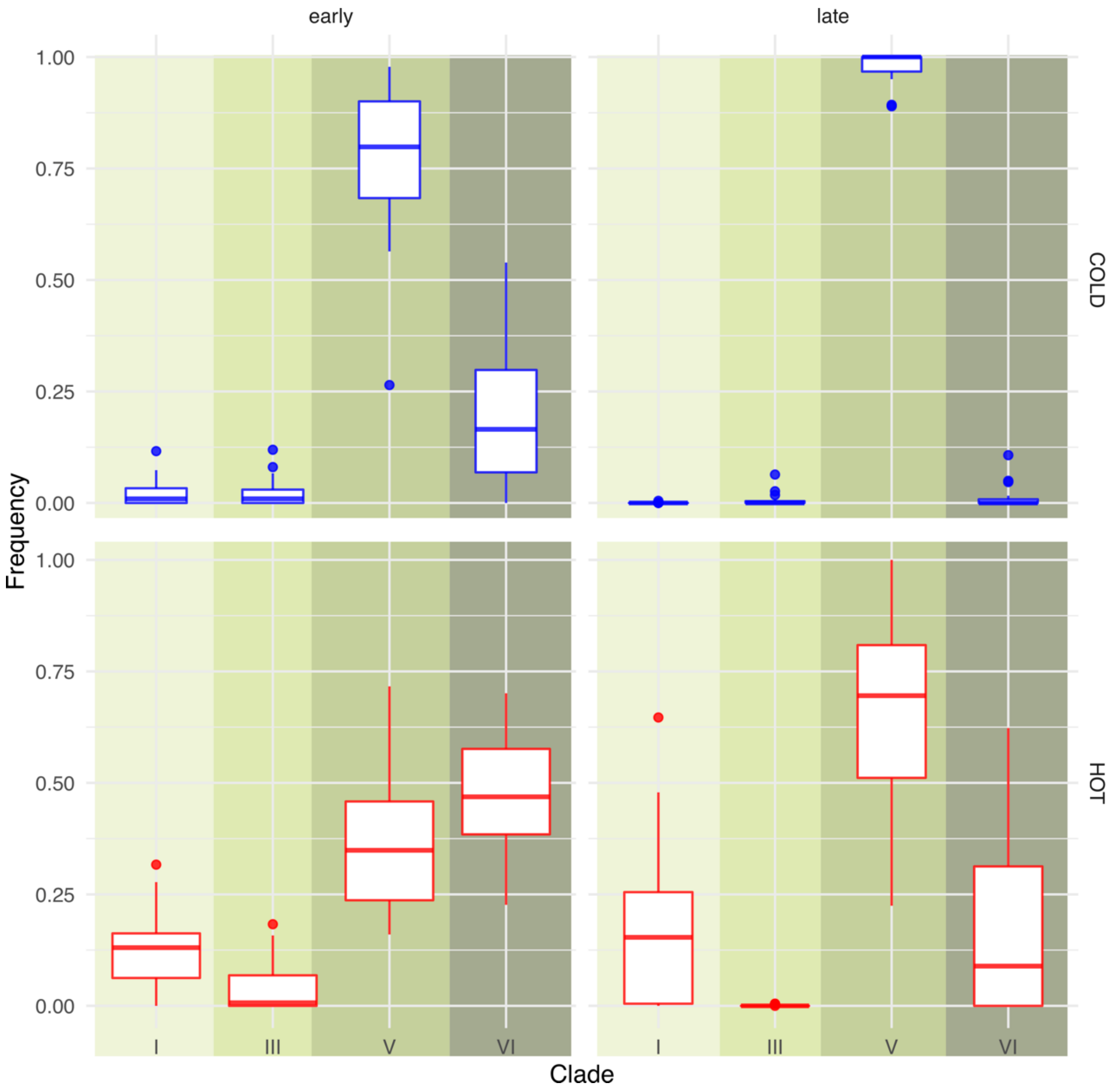

Figure S1: Frequency differences among *Wolbachia* clades. Boxplots (see Fig. 2) show clade frequencies in the cold (blue; upper row) and hot (red; lower row) environment in early (left column; generations < 50 in the cold, < 75 in the hot environment) and late (right column; generations >= 50 in the cold, >=75 in the hot environment). The corresponding *p*-values from pairwise two-sided Wilcox tests are given in Table S3.

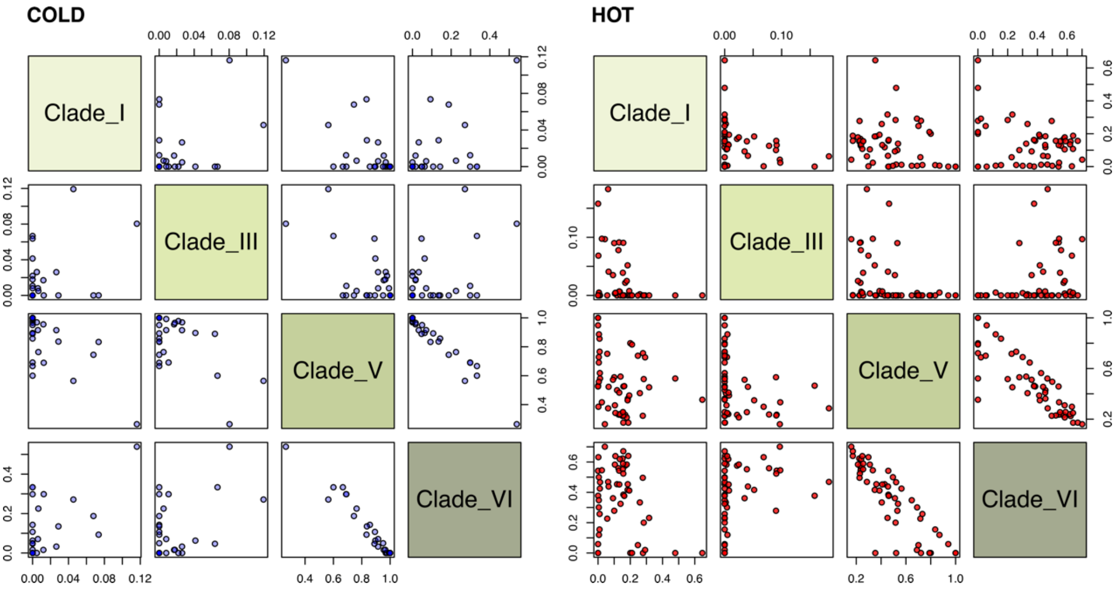

Figure S2: Correlations among *Wolbachia* clade dynamics. Scatterplots show the correlation between the *Wolbachia* clades for the data shown in Fig. 3 of the main text in the cold (left; blue) and hot (right; red) environments. The most conspicuous feature is a strong negative correlation between clades V and VI in both cold (Pearson correlation: –0.98; 95% confidence interval: –1.00, –0.95; *p*-value < 2.2e–16) and hot (Pearson correlation: –0.85; 95% confidence interval: –1.00, –0.77; *p*-value: 5.8e–16).

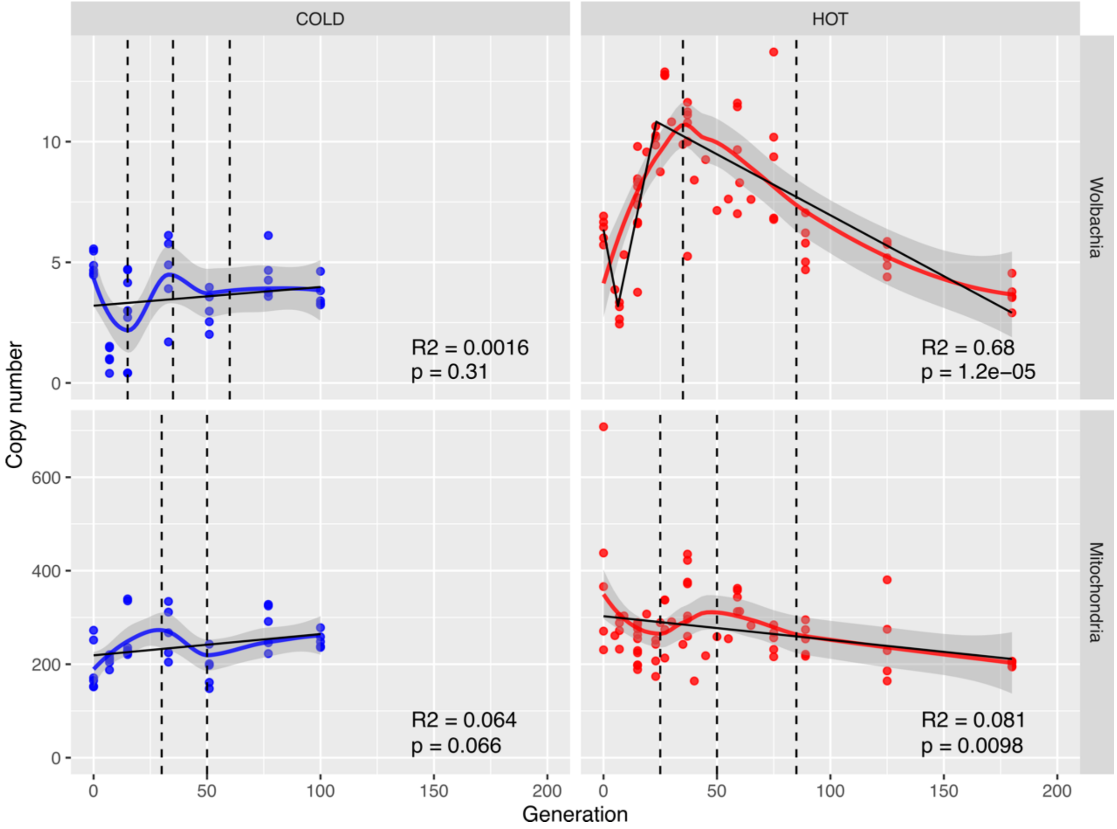

Figure S3: Significance of copy number changes over time. We assess the significance of the features of the *Wolbachia* (upper row) and mitochondria (lower row) dynamics in cold (left column; blue) and hot (right column; red) environments also shown in Figs. 4 and 6 of the main text by fitting optimal piece-wise linear models using the `segmented` package in `R`. Dashed vertical lines indicate possible breakpoints obtained from visual inspection of Figs. 4 and 6, which were used as initial guesses for the procedure. Solid black polygons indicate the final model, with its adjusted R^2 and model p-value shown in the inset text. Only for *Wolbachia* in the hot envrionment were any breakpoints retained by the procedure. Despite the un-segmented linear model being the best model for both *Wolbachia* and mitochondria dynamics in the cold environment, the evidence for a linear trend is not covincing ($p > 0.05$ in both cases). There is some evidence for a weak negative trend for the mitochondria dynamics in the hot environment ($p = 0.01$), but most of the variance remains unexplained by this model (adj. $R^2 = 0.08$). Only the piece-wise linear model for the *Wolbachia* dynamics in the hot environment is both highly significant ($p = 0.00001$) and explains a substantial part of the observed variance (adj. $R^2 = 0.68$), leaving the intermediate peak and subsequent decrease of the *Wolbachia* copy number in the hot environment as the most significant feature of the observed dynamics.

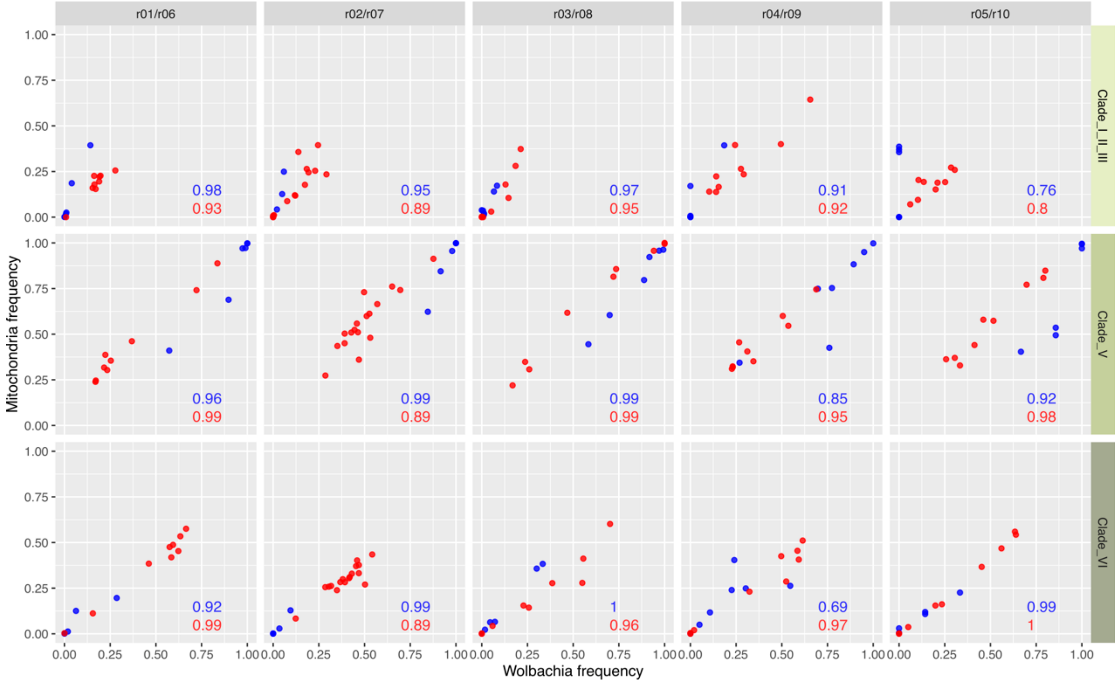

Figure S4: Correlations of mitochondrial and *Wolbachia* dynamics. To quantify the correlation between *Wolbachia* and mitochondria dynamics on the superclade level (Fig. 5 in the main text), we visualize in the panel layout of Fig. 5, the correlation between *Wolbachia* and mitochondria frequencies and calculate the Pearson correlation coefficients (inset text), which is for most super clades and replicates 0.9 or higher.

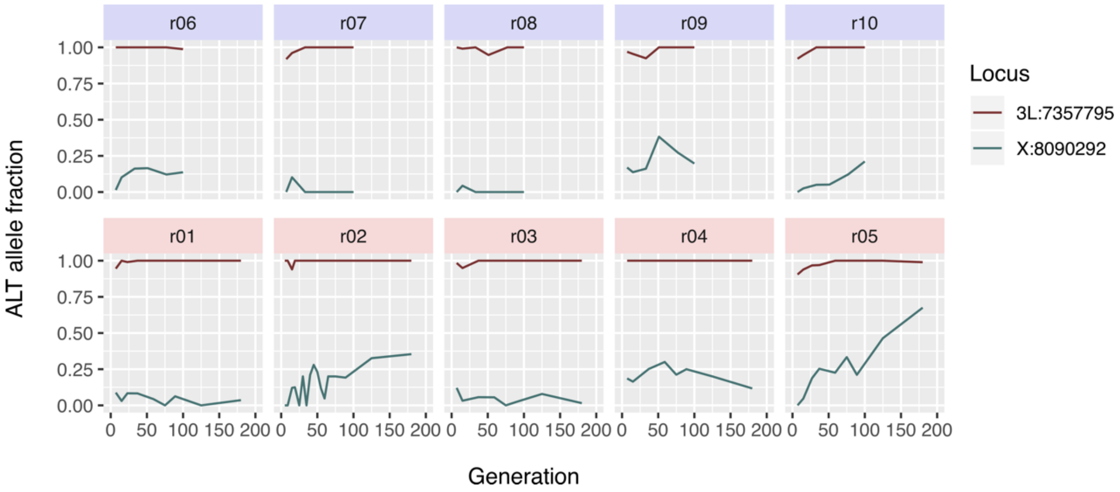

Figure S5: Dynamics of Drosophila C virus resistance alleles. Lines show the frequencies of the ALT allele on loci 3L:7357795 (*pastrel*) (maroon) and X:8090292 (*Ubc-E2h*) (turqois) in the *Drosophila melanogaster* reference genome v6.03 over generations in the cold (upper row) and hot (lower row) environments in five replicates each. Positions were given as 3L:7350895 and X:7984325, respectively, in (Martins et al., 2014) with respect to the v5.38 version of the *dmel* reference genome, and translated with the converter at `http://flybase.org/cgi-bin/coord_converter.pl`. The REF allele on *pastrel* and the ALT allele on *Ubc-E2h* confer virus resistance.

Table S1: *p*-values from pair-wise two-sided Wilcox tests for differences in average copy numbers among *Wolbachia* (a) strains and (b) clades (Fig. 2; *p*-values for clades also labelled in Fig. 2b), and (c) mitochondria superclades (Fig. 6, inset). No multiple-testing adjustments were performed.

a

	x02	x03	x04	w14	x01	x06	x07	w18	w02	x10	x11	w06
x03	1	.	.	.	.	.	.	.	.	.	.	.
x04	0.13333	0.02857	.	.	.	.	.	.	.	.	.	.
w14	0.07143	0.00952	0.11429	.	.	.	.	.	.	.	.	.
x01	0.07143	0.00952	0.11429	0.81818	.	.	.	.	.	.	.	.
x06	0.33333	1.0000	0.13333	0.07143	0.07143	.	.	.	.	.	.	.
x07	0.33333	0.13333	1.000	0.07143	0.07143	0.33333	.	.	.	.	.	.
w18	0.31026	1.0000	0.01281	1.8e–05	1.8e–05	0.46667	0.06410	.	.	.	.	-
w02	0.04444	0.00404	0.00404	0.00067	0.00067	0.04444	0.04444	6.1e–06	.	.	.	.
x10	0.07143	0.00952	0.00952	0.00216	0.00216	0.07143	0.07143	2.8e–07	0.04262	.	.	.
x11	0.33333	0.13333	0.13333	0.07143	0.07143	0.33333	0.33333	0.00256	0.53333	0.07143	.	.
w06	0.07143	0.00952	0.00952	0.00216	0.00216	0.07143	0.07143	2.8e–07	0.00799	0.39394	0.07143	.
x13	0.40000	0.04848	0.00404	0.00067	0.00067	0.40000	0.04444	0.0023	0.72090	0.34499	1.0000	1.0000

b

	Clade_I	Clade_III	Clade_V
Clade_III	0.0832	.	.
Clade_V	0.0075	1.0e–05	.
Clade_VI	1.4e–10	1.0e–06	4.1e–13

c

	Clade_I_II_III	Clade_V
Clade_V	0.89	.
Clade_VI	0.69	0.89

Table S2: Results from a PCR screen for *Wolbachia* infection in the final generations.

Replicate	No. of flies tested	No. of flies negative for *W.*
r01	34 (+ 50)*	0
r02	35	0
r03	31	0
r04	32	0
r05	36	0
r06	33	0
r07	31	1
r08	31	1
r09	30	0
r10	30	0

* An initial 50 flies had been tested earlier only for replicate r01.

Table S3: *p*-values from two-sided Wilcox tests for differences in frequencies among *Wolbachia* clades in early and late generations and (a) cold and (b) hot environments (Fig. S1). No multiple-testing adjustments were performed.

a

	Clade_I.early	Clade_I.late	Clade_III.early	Clade_III.late	Clade_V.early	Clade_V.late	Clade_VI.early
Clade_I.late	6.1e-06	.	.	.	.	.	.
Clade_III.early	0.92600	8.0e-06	.	.	.	.	.
Clade_III.late	0.00172	0.53930	0.00201	.	.	.	.
Clade_V.early	3.3e-09	6.7e-09	3.3e-09	6.7e-09	.	.	.
Clade_V.late	1.7e-06	2.5e-06	1.7e-06	2.5e-06	2.4e-05	.	.
Clade_VI.early	0.00011	4.7e-08	0.00011	9.3e-07	1.0e-07	1.7e-06	.
Clade_VI.late	0.01058	0.36688	0.01058	0.68267	6.7e-09	2.5e-06	4.6e-06

b

	Clade_I.early	Clade_I.late	Clade_III.early	Clade_III.late	Clade_V.early	Clade_V.late	Clade_VI.early
Clade_I.late	0.86288	.	.	.	.	.	.
Clade_III.early	1.6e-06	0.01931	.	.	.	.	.
Clade_III.late	2.7e-11	3.4e-05	4.5e-05	.	.	.	.
Clade_V.early	5.9e-13	3.7e-05	< 2e-16	9.9e-15	.	.	.
Clade_V.late	2.9e-09	9.1e-07	1.5e-09	6.8e-08	2.8e-05	.	.
Clade_VI.early	< 2e-16	4.8e-08	< 2e-16	9.9e-15	0.00095	0.00342	.
Clade_VI.late	0.82021	0.96800	0.13094	0.00672	0.00127	4.0e-06	6.7e-06
